# Home-Based Monitoring and Telemonitoring of Complicated Pregnancies: Nationwide Cross-Sectional Survey of Current Practice in the Netherlands

**DOI:** 10.2196/18966

**Published:** 2020-10-28

**Authors:** Josephus F M van den Heuvel, Samira Ayubi, Arie Franx, Mireille N Bekker

**Affiliations:** 1 Department of Obstetrics University Medical Center Utrecht University Utrecht Netherlands; 2 Department of Obstetrics and Gynaecology Erasmus Medical Center Erasmus University Rotterdam Netherlands

**Keywords:** mobile health, telemonitoring, pregnancy complications, digital health, telemedicine

## Abstract

**Background:**

Daily monitoring of fetal and maternal conditions in complicated pregnancies leads to recurrent outpatient visits or (prolonged) hospitalization. Alternatives for hospital admissions include home-based monitoring with home visits by professionals or telemonitoring with self-measurements performed by pregnant women and uploaded for in-clinic assessment. For both alternatives, cardiotocography and blood pressure measurement can be performed at home. It is unknown to what extent, for which reasons, and for which pregnancy complications these strategies are used.

**Objective:**

This study aims to assess the current practice and attitudes concerning home-based monitoring (with daily home visits by professionals) and telemonitoring (using devices and the internet for daily self-recorded measurements) in high-risk pregnancies requiring maternal and fetal monitoring in the Netherlands.

**Methods:**

This nationwide cross-sectional study involved sending a web-based survey to the obstetrics departments of all 73 hospitals in the Netherlands to be answered by 1 representative dedicated to pregnancy monitoring per hospital. The primary outcome was the provision of home-based monitoring or telemonitoring using cardiotocography between 1995 and 2018. The survey further addressed perspectives regarding the use of home-based monitoring and telemonitoring, including (contra)indications, advantages, and disadvantages for pregnant women and clinicians.

**Results:**

The response rate for the provision of either home-based monitoring or telemonitoring was 100%. In 2018, 38% (28/73) of centers in the Netherlands offered either home-based monitoring or telemonitoring or both to pregnant women with complications. Home-based monitoring was offered in 26% (19/73) of the centers; telemonitoring, in 23% (17/73); and both in 11% (8/73). Telemonitoring was first offered in 2009, increasing from 4% (3/73) of hospitals in 2014 to 23% (17/73) in 2018. Responses were received from 78% (57/73) of the invited hospitals and analyzed. Of all 17 centers using telemonitoring, 59% (10/17) did not investigate perinatal outcomes, safety, and patient satisfaction prior to implementation. Other (6/17, 35%) telemonitoring centers are participating in an ongoing multicenter randomized clinical trial comparing patient safety, satisfaction, and costs of telemonitoring with standard hospital admission. Home-based monitoring and telemonitoring are provided for a wide range of complications, such as fetal growth restriction, pre-eclampsia, and preterm rupture of membranes. The respondents reported advantages of monitoring from home, such as reduced stress and increased rest for patients, and reduction of admission and possible reduction of costs. The stated barriers included lack of insurance reimbursement and possible technical issues.

**Conclusions:**

Home-based monitoring is provided in 26% (19/73) and telemonitoring, in 23% (17/73) of hospitals in the Netherlands to women with pregnancy complications. Altogether, 38% (28/73) of hospitals offer either home-based monitoring or telemonitoring or both as an alternative to hospital admission. Future research is warranted to assess safety and reimbursement issues before more widespread implementation of this practice.

## Introduction

Pregnancies with complications need close antenatal surveillance. Although 7-10 antenatal consultations are recommended in uncomplicated pregnancies, complications result in recurrent outpatient visits or hospital admission [1]. These complications include fetal growth restriction, pre-eclampsia, and preterm prelabor rupture of membranes (prevalence of 3%-7%, 1%-3%, and 1%-5%, respectively) [2-4]. Daily monitoring with cardiotocography (CTG), blood pressure measurements, and/or urine and blood analysis is recommended in international guidelines to assess maternal and fetal conditions in high-risk pregnancies [5-7]. Ultimately, hospitalization is indicated in up to 11% of all pregnancies, usually extending to delivery and the postpartum period [5-7]. Antenatal admissions pose psychological stress to pregnant women because of separation from family and home, lack of activity, and feelings of uncertainty [8,9]. In addition, hospital admissions increase health care costs and workload, especially in high-income countries that are already experiencing difficulties as a result of professional staff shortage [10].

**Figure 1:**
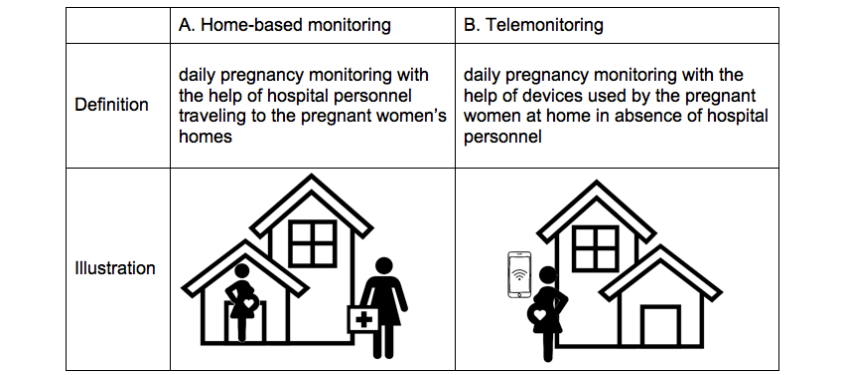
Definition and illustration of (A) home-based monitoring and (B) telemonitoring in pregnancy.

Since 1990, obstetrics departments in the Netherlands have been providing domiciliary care or “home-based monitoring” to women with high-risk pregnancies. As an alternative to clinical admission, home-based monitoring involves daily home visits from hospital-employed midwives or nurses for pregnant women with complications. Medical tests, including CTG, are performed at home, and the results are discussed with a supervising gynecologist (Figure 1A). Multiple randomized trials have proved that home-based monitoring with home visits is feasible and safe with regard to perinatal outcome [11-14]. Although these trials demonstrated satisfactory outcomes for both mother and child, the daily visits were also found to be time-consuming and, therefore, expensive.

The use of digital health for remote monitoring in pregnancy care is increasingly popular, as pregnant women are frequent users of smartphones, the internet, and health apps [15]. *Tele*monitoring is a relatively new approach in high-risk pregnancy and is recognized as an alternative to hospital admission or home-based monitoring with prenatal home visits. After training participants, daily measurements of blood pressure and CTG are self-recorded by the patient at home and sent via Bluetooth or WiFi to a secured digital platform. Using an internet connection, the data are integrated in the electronic patient file (Figure 1B). Patients are contacted by their clinician on a daily basis to discuss the presence of symptoms, tests results, and future management. Multiple telemonitoring platforms for remote CTG have been evaluated in prospective studies, and their feasibility, usability, accuracy of tracings, and acceptability by patients and clinicians are proven [15].

In general, digital health has the potential to improve access to care, disease monitoring, and patient satisfaction while reducing health care costs due to a reduction in visits and admissions. Currently, the clinical evidence for telemonitoring using CTG in complicated pregnancies is too scarce to support hypotheses regarding its effects on perinatal outcome, safety, patient preference, and costs.

A number of hospitals in the Netherlands currently provide either home-based monitoring or telemonitoring or both to women with high-risk pregnancies. It is unknown to what extent, for which reasons, and for which pregnancy complications these strategies are used. This information is relevant for clinicians planning to use a telemonitoring strategy in prenatal care. The aim of this nationwide survey study is to determine the number of hospitals in the Netherlands that provide home-based monitoring or telemonitoring or both, and to identify the current practice of out-of-hospital care in high-risk pregnancies.

## Methods

We conducted a nationwide cross-sectional study using a web-based survey amongst obstetrics care professionals. All hospitals with pregnancy and childbirth care departments in the Netherlands (N=73) were invited to participate in our survey. They were asked to appoint one of their obstetrics care professionals dedicated to (remote) pregnancy monitoring as the department representative to answer the questions on behalf of the hospital. After receiving additional information about the purpose of the study, access was provided to our web-based survey. The survey link was sent via email in November 2018 and followed by a maximum of 3 email reminders. Nonrespondents were contacted once more by phone to answer the principal question “Does your center currently offer home-based monitoring or telemonitoring in pregnancy?”

The survey was self-developed and based on expert knowledge of home-based monitoring and telemonitoring in the Netherlands. A professor of obstetrics, a perinatologist, a hospital-based midwife, and 2 researchers, all with extensive experience in home-based monitoring of high-risk pregnancies, were involved in its development. It contained a maximum of 44 questions depending on whether home-based monitoring or telemonitoring was offered. The open and multiple-choice questions addressed 4 domains: (1) Basic demographics of the respondent, (2) home-based monitoring, (3) telemonitoring, and (4) advantages and disadvantages of home-based monitoring and telemonitoring, as perceived by the respondent (see Multimedia Appendix 1).

The survey sought information on the total number of births per year in order to compare the hospitals with reference to size. Regarding the provision of home-based monitoring or telemonitoring, the starting year and, if applicable, year of discontinuation were queried. We defined our study period from 1995 to 2018. Questions regarding indications, management protocols, and (dis)advantages of the strategies were asked bearing in mind the centers’ practice for the year 2018. The introduction to our survey defined home-based monitoring as “daily pregnancy monitoring with help of hospital personnel traveling to the pregnant women’s homes.” Telemonitoring was defined as “daily pregnancy monitoring with the help of devices used by pregnant women at home in the absence of hospital personnel” (Figure 1). Simple descriptive statistics were used to describe the results. No ethical approval was required for this study** **because actual patients were not involved**. **

## Results

### Current Provision of Home-Based Monitoring and Telemonitoring in High-Risk Pregnancy

In 2018, 73 hospitals in the Netherlands provided pregnancy and childbirth care. The principle question, namely “Does your center currently offer home-based monitoring or telemonitoring in pregnancy?” was answered by all 73 invitees, resulting in a response rate of 100%.

In 2018, 26% (19/73) hospitals offered home-based monitoring with home visits by an obstetrics professional (nurse or midwife) for women with high-risk pregnancies. Nationwide, 23% (17/73) of hospitals offered telemonitoring in 2018 to women with high-risk pregnancies (Table 1). Moreover, 11% (8/73) of centers reported offering both home-based monitoring with home visits and telemonitoring to their patients.

**Figure 2:**
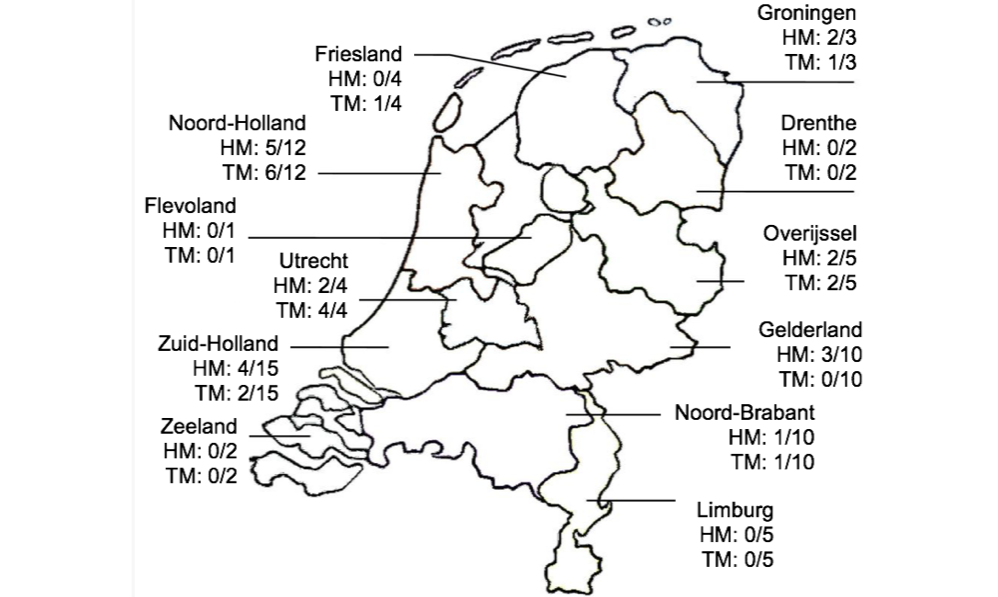
Geographic distribution of home-based monitoring and telemonitoring in 12 provinces of the Netherlands (N=73). HM: hospitals with home-based monitoring/all hospitals in this province; TM: hospitals with telemonitoring/all hospitals in this province.

In obstetrics departments with ≤1000 births per year, home-based monitoring and telemonitoring is limited to 0 and 1 centers, respectively. As for the different types of hospitals, 8 of 9 Dutch tertiary care hospitals with a neonatal intensive care unit facility currently offer home-based monitoring or telemonitoring or both for high-risk pregnancy management. The geographic distribution of hospitals with home-based monitoring and telemonitoring is displayed in Figure 2 for all 12 provinces of the Netherlands.

**Table 1:** Number of hospitals offering home-based monitoring and telemonitoring for high-risk pregnancies in 2018 in relation to the number of births per hospital per year.

Number births per hospital per year	Number of hospitals (N=73), n (%)^a^	Home-based monitoring (n=19), n (%)^a^	Telemonitoring (n=17), n (%)^a^
0-1000	15 (21)	0 (0)	1 (7)
1001-2000	35 (48)	9 (26)	8 (23)
2001-3000	21 (29)	9 (43)	6 (29)
>3000	2 (2)	1 (50)	2 (100)

^a^Percentages in these columns are based on the number of hospitals in each row.

**Figure 3:**
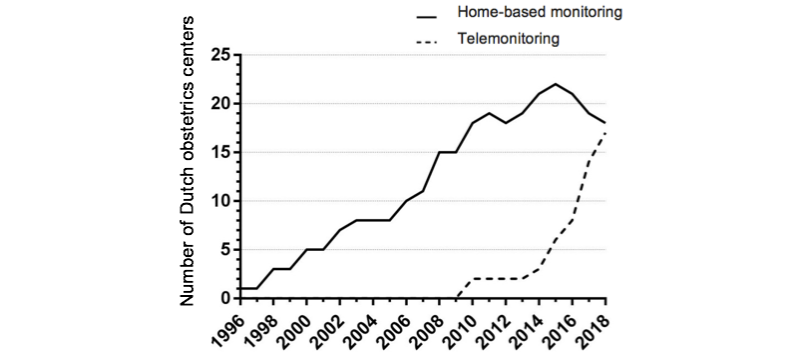
Trend graph of obstetrics departments offering home-based monitoring and telemonitoring for high-risk pregnancies in the Netherlands.

For the studied period of 1995-2018, the trend line in Figure 3 shows that home-based monitoring in pregnancy has been offered since the mid-1990s. Most of these centers continued offering daily home visits over a longer period of time, reaching a peak in 2015. After the introduction of pregnancy telemonitoring in 2009, the trend line for telemonitoring use shows a steep increase from 2014 onwards, from 4% (3/73) of centers in 2014 to 23% (17/73) in 2018. This increase in number is accompanied by a slight drop in home-based monitoring provision.

### Survey Results

#### Respondents’ Characteristics

Of the total 73 invited hospitals, 57 participated in the web-based survey (response rate 78%). Of these respondents, 45% (26/57) worked in a teaching hospital, 39% (22/57) served in a nonteaching hospital, and 16% (9/57) worked in an tertiary care hospital with a neonatal intensive care unit. Moreover, 14% (8/57) reported 0-1000 births per year, 51% (29/57) had 1001-2000 births per year, 32% (18/57) recorded 2001-3000 births per year, and 3% (2/57) reported over 3000 births per year.

#### Declining Trend of Home-Based Monitoring

The results showed that 11% (6/57) of centers offered home-based monitoring in the years between 1995 and 2018 but stopped performing pregnancy monitoring with home visits. The median number of years of home-based monitoring provision was 7.5 years (range 2-18 years). Several reasons were provided for the discontinuation, such as very few possible candidates (3/6, 50%), problems with staff capacity (3/6, 50%), financial capacity issues (2/6, 33%), and switching over to telemonitoring without home visits (2/6, 33%).

Moreover, 42% (8/19) of hospitals with home-based monitoring considered switching to telemonitoring, stating that the latter provides higher patient satisfaction and does not require hospital staff to visit patients at home. Interestingly, 16% (3/19) of hospitals providing home-based monitoring did not change to telemonitoring because they are satisfied with their current home-based monitoring strategy. They stated that telemonitoring allows neither daily direct clinical assessment of the patient by a nurse/midwife nor the ability to monitor twin pregnancies.

#### Evaluation of Use

In 63% (12/19) of hospitals offering home-based monitoring, implementation of such monitoring was not preceded by a center-specific evaluation phase. However, home-based monitoring in these centers started mainly after the publication of the findings of 2 Dutch trials, which concluded positively about its patient safety and effects on satisfaction of care [7,8].

As for telemonitoring, 35% (6/17) of centers are participating in a multicenter randomized controlled trial comparing clinical hospital admission with telemonitoring in pregnancies requiring daily fetal monitoring. The aim of this trial is to compare patient safety, user satisfaction, and cost-effectiveness; its protocol can be found elsewhere [16]. The remaining 59% (10/17) of centers reported they neither participated in nor started evaluation of use of this novel strategy with daily self-measurements prior to implementation in complicated pregnancies in their centers.

#### Indications and Management in Home-Based Pregnancy Monitoring

Responding centers implementing either home-based monitoring or telemonitoring reported similar lists of pregnancy complications, which they considered eligible for daily monitoring outside their hospital (Table 2). Both fetal growth restriction and preterm rupture of membranes are considered eligible for home-based monitoring as well as telemonitoring in every center.

All (18/18, 100%) home-based monitoring centers reported that at home, midwives or nurses measure patients’ blood pressure levels and perform CTGs during their visits. Fetal condition monitoring of both singleton and twin pregnancies using CTG is possible in 83% (15/18) of centers. Additionally, urine analysis (13/18, 72%), venous blood sampling (12/18, 67%), and medication administration (4/18, 22%) can be performed by professionals at home. This is in contrast to telemonitoring centers, where only blood pressure monitoring and CTG are performed by patients themselves at home.

Hospitals with either home-based monitoring or telemonitoring also reported on the following patient-specific contraindications for home-based monitoring: inability to follow instructions or difficulty in understanding the system (26/28, 93%), long home-to-hospital distance (25/28, 89%), existing antepartum hemorrhage (20/28, 71%), and vulnerable home situation or social issues experienced by the patient (14/28, 50%). Other mentioned general contraindications were gestational age<25 weeks and preterm premature rupture of membranes without engaged fetal head or breech. To ensure the safety of the patients by minimizing travel time if complications occur, the respondents clarified that patients must reside within a distance of 30-35 km from their hospital.

**Table 2:** Indications for home-based monitoring (n=19) and telemonitoring (n=17) in high-risk pregnancies.

Indications	Home-based monitoring centers, n (%)	Telemonitoring centers, n (%)
Fetal growth restriction	19 (100)	17 (100)
Preterm premature rupture of membranes	19 (100)	17 (100)
Prolonged prelabor rupture of membranes at term	5 (26)	2 (12)
Isolated oligohydramnios	10 (53)	4 (24)
Reduced fetal movement	15 (79)	15 (88)
Fetal anomalies requiring fetal monitoring	9 (47)	3 (18)
(Adverse) Obstetrics patient history^a^	16 (84)	15 (88)
Hypertensive pregnancy disorders	15 (79)	10 (59)
Cholestasis of pregnancy	14 (74)	5 (29)
Other maternal comorbidities^b^	11 (58)	4 (24)
Social or psychological distress	11 (58)	5 (29)

^a^For instance, intrauterine fetal death in a previous pregnancy.

^b^For instance, (gestational) diabetes mellitus, kidney disease, and cardiac disease requiring maternal monitoring.

#### Reported Advantages and Disadvantages of Home-Based Monitoring and Telemonitoring

The most frequently addressed advantages of home-based monitoring and telemonitoring for the patients, as perceived by the respondents, include more patient comfort and less emotional burden of hospitalization for the patient as they continue with daily (family) life and activities as much as possible. Other frequently mentioned advantages are summarized in Table 3.

Possible disadvantages of home-monitoring and telemonitoring for the patient include the possibility of a delay in providing help in case of an emergency or acute problem, because the patient is not physically present in the hospital. Technical and security issues regarding the devices are also mentioned (Table 3).

The respondents reported a number of perceived benefits of home-based monitoring and telemonitoring for the health care provider, the most important being the reduction in the number of admissions, which in turn may lower health care costs (45/57, 79%) and reduce the burden on hospital personnel (26/57, 46%). The most common disadvantages of home-based monitoring and telemonitoring for the clinicians are costs and reimbursement (38/57, 67%) and inability to conduct direct patient assessments (18/57, 31%). For home-based monitoring specifically, the most serious disadvantage was lack of sufficient obstetrics personnel to make home visits (22/57, 39%).

**Table 3:** Advantages and disadvantages of home-based monitoring and telemonitoring for patients according to the respondents (n=57).

Advantages or disadvantages		Values
**Advantages, n (%)**		
	Improved patient comfort	40 (70)
	Reduced (emotional) burden of admission	35 (61)
	Reduced stress/more rest	25 (44)
	Better patient autonomy	21 (37)
	Higher patient satisfaction	8 (14)
	Higher patient safety	7 (12)
	Reduced over-medicalization during pregnancy	2 (4)
**Disadvantages, n (%)**		
	Possible delay in providing help during emergencies or acute problems	38 (67)
	No direct communication with the consulting gynecologist	18 (31)
	Patients’ inability to conduct CTG^a^ at home	13 (23)
	Technical issues	17 (30)
	Inability to follow instructions	12 (21)

^a^CTG: cardiotocography.

#### Number of High-Risk Pregnant Women Managed From Home

All (19/19, 100%) of home-based monitoring centers reported monitoring a minimum of 745 to a maximum of 1140 patients with a singleton pregnancy via home visits in 2018.

All of the telemonitoring centers (17/17, 100%) reported monitoring a minimum of 400 to a maximum of 725 patients with a singleton pregnancy via remote monitoring devices in 2018.

## Discussion

### Main Findings

Our survey results show the current practice in the Netherlands regarding the use of home-based monitoring and telemonitoring in high-risk pregnancies. In 1995, pregnancy monitoring with daily home visits was available in only a few obstetrics hospitals; currently, it is used by 26% (19/73) of all hospitals in the Netherlands. The last 5 years have witnessed a steep increase in the provision of telemonitoring; as of 2018, it was used in 23% (17/73) of obstetrics departments. Furthermore, almost half (8/19, 42%) of the hospitals with home-based monitoring considered switching to telemonitoring using self-measurement of fetal and maternal parameters. For the telemonitoring centers, 59% (10/17) did not evaluate the use of this digital health strategy with daily self-measurements prior to implementation in their centers. Moreover, 35% (6/17) of centers are currently participating in an ongoing trial to compare traditional hospital admission and telemonitoring with regard to patient safety, satisfaction, and costs.

In 2018, 1145-1865 pregnant women were monitored from home with home visits or telemonitoring after diagnosis of 1 or more complications.

### Strengths and Limitations

Our study involved a nationwide survey with a high response rate and included both secondary and tertiary referral centers, teaching and nonteaching centers, and a wide size range of units according to annual birth numbers. The responses to the survey depended on the voluntary participation of the invited hospitals, which could have led to selection bias. Furthermore, the collected data were self-reported and hence subjective. Some of the results on the impact of remote monitoring were based on estimations by the respondents, which may limit the validity of the conclusions. Evaluations of the characteristics of pregnant women, relevant clinical outcomes (including safety), and user experiences are critical for future health care improvements using mobile health monitoring. However, this study was not devised to evaluate these outcomes, which might be considered a limitation.

### Interpretation

The level of application of digital health in prenatal care is evident, with pregnancy telemonitoring being one of the most promising additions to new care models [9,15,17]. The respondents of our survey identified important (perceived) advantages of telemonitoring: improved patient-friendly care in response to their needs, increased patient satisfaction and autonomy, and reduced over-medicalization. These results are in line with those of previous research on patient experiences with digital health [15,18,19]. Furthermore, obstetrics care professionals underscore the importance of digital health in pregnancy care. For instance, a survey study conducted in Belgium concluded that 80% (28/35) of midwives and 67% (6/9) of obstetricians who used remote blood pressure monitoring in pregnancy perceived digital technologies to be an important component of prenatal monitoring [20]. Moreover, a survey amongst 89 German physicians concluded that nearly 70% considered apps for pregnancy monitoring reasonable [21]. Other reported advantages in favor of telemonitoring are the reductions in the number of admissions and the burden on hospital personnel [18,19]. Staff shortages are also driving a shift from hospital- to home-based care.

In the Netherlands, approximately 170,000 children are delivered per year from both uncomplicated and complicated pregnancies. We estimated earlier that 11% of pregnant women need antenatal hospital admission because of complications, which equals to 18,700 women yearly [2-4]. Using our respondents’ results, we calculated that 1145-1865 pregnant women were monitored from home in 2018. This number indicates that roughly 6%-10% of antenatal hospital admissions were replaced by home-based monitoring or telemonitoring in 2018. Although exact numbers on the length of hospitalization during high-risk pregnancies are lacking, we can use these values to estimate the possible impact of home-based and telemonitoring on admissions during pregnancy. If home-based monitoring or telemonitoring services in pregnancy were to replace 5 days or nights of hospitalization per pregnant woman, the number of hospital admission days would reduce by 5,725-9,325 annually.

Studies on telemonitoring implementation using patient-recorded daily CTG are limited. Despite the inadequate knowledge of the effects of pregnancy telemonitoring on perinatal outcomes, patient experiences, and cost-effectiveness, this study shows that telemonitoring is becoming increasingly popular in the Netherlands. Although not mentioned by our respondents, legal concerns such as third party control and use of data limit the widespread use of digital health interventions. In the Netherlands, external companies providing devices, software, and storage of patient data for telemonitoring must provide certain data security assurances. Evidence from clinical trials and health technology assessments will help to better estimate the exact budgetary impact from several different (ie, societal, insurance, and hospital) perspectives. The costs involved in the development, use, and maintenance of the devices, as well as the manner in which they are imbedded in current practice, should also be calculated to assess the added value of pregnancy telemonitoring. Our survey respondents reported challenges with reimbursement, since no insurance coverage for pregnancy telemonitoring exists in the Netherlands. Financial issues were another primary reason mentioned by our respondents (especially the smaller obstetrics units) to not offer either home-based monitoring or telemonitoring. It is well known that insurance companies only cover well-researched digital health interventions in accordance with their economic evaluations [22,23]. To compare daily telemonitoring at home with traditional hospital care for complicated pregnancies, a multicenter randomized controlled trial, HOTEL (HOspital admission versus TELemonitoring in high risk pregnancy), is currently recruiting participants in 6 Dutch hospitals [16]. This trial aims to compare both strategies with regard to perinatal outcomes, patient satisfaction, and cost-effectiveness.

### Recommendations for Research and Practice

Our survey study provides information about the current practice and trends in the Netherlands regarding home-based monitoring and telemonitoring in perinatal care. More detailed information on the barriers and facilitators, from both the patients’ and health care providers’ viewpoints, may help develop other innovative strategies in perinatal care. However, evidence on the medical outcomes and patient safety with telemonitoring is still lacking, and more information is required before implementing such innovations in the target population. We must expand our knowledge of these forms of care in order to continue moving forward with digital health innovations. Consensus on the implementation and research agenda can pave the road to the widespread use of digital health services. Additional trials and stakeholders’ views of digital health care are needed to develop insurance reimbursement systems for such remote monitoring innovations in pregnancy.

### Conclusion

In 2018, 26% (19/73) of hospitals in the Netherlands offered home-based monitoring and 23% (17/73) offered telemonitoring to their patients with pregnancy complications. These increasingly popular forms of home-based care allow an increasing number of pregnant women in need of daily monitoring to stay at home and avoid hospital admission. Additionally, the survey respondents shed light on multiple possible advantages and disadvantages of home-based monitoring and telemonitoring in pregnancy. These results can contribute to future evaluations of digital innovations in pregnancy care, as further research on their safety, experience, and cost-effectiveness is warranted before more widespread implementation.

## Appendix

Multimedia Appendix 1 Overview of the survey used in this study.

## Abbreviations

CTG: cardiotocography
HOTEL: HOspital admission versus TELemonitoring in high risk pregnancy
