# Supplementary material for: Home-Based Monitoring and Telemonitoring of Complicated Pregnancies: Nationwide Cross-Sectional Survey of Current Practice in the Netherlands
Source: JMIR Mhealth Uhealth. 2020 Oct 28;8(10):e18966. doi: 10.2196/18966 (PMC7657725; doi:10.2196/18966)
Supplement: Multimedia Appendix 1 [file mhealth_v8i10e18966_app1.docx]

For all hospitals:

1. Your hospital lies in which province of the Netherlands?

Options: Groningen, Friesland, Drenthe, Overijssel, Flevoland, Gelderland, Utrecht, Noord-Holland, Zuid-Holland, Zeeland, Noord-Brabant, Limburg

1. Your hospital is a

- Secondary obstetric care center
- Tertiary obstetric care center (With NICU facility)

1. Is your hospital a teaching hospital?

Options: yes, no

1. What is the estimated number of deliveries per year in your hospital

Options: 0-1000, 1001-2000, 2001-3000, 3001 or more

1. What is your function?

Options: gynaecologist, midwife, nurse, other

1. Does your hospital currently provide home monitoring
   Options: yes; no, but we did in the past; no
2. Does your hospital currently provide tele-monitoring
   Options: yes; no, but we did in the past; no

For hospitals with current home monitoring:

1. Since what year does your hospital provide home monitoring

Open question

1. Does your hospital work with a local guideline regarding pregnancy home monitoring
   Options: yes, no
2. Did your hospital conduct a local evaluation of home monitoring regarding patient safety before implementation?

Options: yes, no,

1. Did your hospital conduct a local evaluation of home monitoring regarding patient experiences before implementation?

Options: yes, no

1. What medical checks are performed at home by hospital personnel? Multiple options possible
   Options: physical examination, CTG, blood pressure, temperature, urine sampling, draw blood, give medication (for example corticosteroids), other…
2. What groups of high risk pregnancy are eligible for home monitoring in your hospital? Multiple options are possible.

Options: FGR, PPROM, preeclampsia, decreased fetal movements, (gestational) hypertension, cholestasis of pregnancy, fetal anomalies requiring surveillance, (gestational) diabetes mellitus, prolonged prelabor rupture of membranes at term, isolated oligohydramnios, (adverse) obstetric patient history, social or psychological stress, other maternal co-morbidity, other..

1. Do you monitor multiple pregnancies using home monitoring

Options; yes, no

1. What contra-indications of home monitoring do you use? Multiple options possible

Options: antepartum haemorrhage, long home-to-hospital distance, non-compliance to agreements, other

1. What is the maximum distance for home monitoring eligibility?

Open question

1. What is the minimum gestational age for home monitoring eligibility?

Open question

1. What is the estimated number of singleton pregnancies that are monitored with home monitoring per year in your hospital?

Options: 0-5, 5-10, 10-25, 25-50, 50-75, 75-100, >100

1. What is the estimated number of multiple pregnancies that are monitored with home monitoring per year in your hospital?

Options: 0-5, 5-10, 10-25, 25-50, 50-75, 75-100, >100

1. What is the estimated number of days that each high-risk pregnant woman is monitored at home?

Options: 0-5, 5-10, 10-15, >15

For hospitals that discontinued home monitoring:

1. From what year to what year did your hospital provide home monitoring?

Open question

1. Why did your hospital stop providing home monitoring? Multiple options possible.
   Options: small number of possible candidates; patients were not interested; obstetric professionals were not interested; not enough staff capacity for home visits; problems with financial capacity to continue home visits; we switched to TELEmonitoring without home visits.
2. Is your hospital planning to re-start with home monitoring?

Options Yes, why; no, why not

For hospitals without current home monitoring:

1. Why is your hospital currently not providing home monitoring?

Options: Options: small number of possible candidates; patients were not interested; obstetric professionals were not interested; not enough staff capacity for home visits; problems with financial capacity to continue home visits; we provide TELEmonitoring without home visits; other..

1. Is your hospital planning to start providing home monitoring?

Options Yes, why; no, why not

For hospitals with current telemonitoring:

1. Since what your did your hospital stat telemonitoring:

Open question

1. What medical check are performed by the pregnant woman herself at home? Multiple options possible.

Options: CTG, blood pressure, temperature, urine analysis, other..

1. Does your hospital work with a local guideline regarding pregnancy telemonitoring
   Options: yes, no
2. Did your hospital conduct a local evaluation of telemonitoring regarding patient safety before implementation?

Options: yes, no,

1. Did your hospital conduct a local evaluation of telemonitoring regarding patient experiences before implementation?

Options: yes, no

1. What groups of high risk pregnancy are eligible for telemonitoring in your hospital? Multiple options are possible.

Options: FGR, PPROM, preeclampsia, decreased fetal movements, (gestational) hypertension, cholestasis of pregnancy, fetal anomalies requiring surveillance, (gestational) diabetes mellitus, prolonged prelabor rupture of membranes at term, isolated oligohydramnios, (adverse) obstetric patient history, social or psychological stress, other maternal co-morbidity, other..

1. Do you monitor multiple pregnancies using telemonitoring

Options; yes, no

1. What contra-indications of telemonitoring do you use? Multiple options possible

Options: antepartum haemorrhage, long home-to-hospital distance, non-compliance to agreements, other

1. What is the maximum distance for telemonitoring eligibility?

Open question

1. What is the minimum gestational age for home monitoring eligibility?

Open question

1. What is the estimated number of singleton pregnancies that are monitored with telemonitoring per year in your hospital?

Options: 0-5, 5-10, 10-25, 25-50, 50-75, 75-100, >100

1. What is the estimated number of multiple pregnancies that are monitored with telemonitoring per year in your hospital?

Options: 0-5, 5-10, 10-25, 25-50, 50-75, 75-100, >100

1. What is the estimated number of days that each high-risk pregnant woman is monitoredusing telemonitoring at home?

Options: 0-5, 5-10, 10-15, >15

For hospitals without current telemonitoring:

1. Why is your hospital currently not providing telemonitoring?

Options: Options: small number of possible candidates; patients were not interested; obstetric professionals were not interested; not enough staff capacity for home visits; problems with financial capacity to continue home visits; we provide home monitoring with home visits; other..

1. Is your hospital planning to start providing telemonitoring?

Options Yes, why; no, why not

For all hospitals:

1. From the viewpoint of the obstetric care professional: can you mention 3 advantages of monitoring risk pregnancies from home?

Open question

1. From the viewpoint of the obstetric care professional: can you mention 3 disadvantages of monitoring risk pregnancies from home?
2. From the viewpoint of the pregnant women: can you mention 3 advantages of monitoring risk pregnancies from home?
3. From the viewpoint of the pregnant women: can you mention 3 disadvantages of monitoring risk pregnancies from home?
